# Metabolic Reprogramming and Cell Adhesion in Acute Leukemia Adaptation to the CNS Niche

**DOI:** 10.3389/fcell.2021.767510

**Published:** 2021-12-10

**Authors:** Nitesh D. Sharma, Esra’a Keewan, Ksenia Matlawska-Wasowska

**Affiliations:** ^1^ Department of Pediatrics, Division of Hematology-Oncology, University of New Mexico Health Sciences Center, Albuquerque, NM, United States; ^2^ Comprehensive Cancer Center, University of New Mexico, Albuquerque, NM, United States

**Keywords:** central nervous system, CNS, meninges, cell adhesion, metabolism, acute lymphoblastic leukemia, acute myeloid leukemia

## Abstract

Involvement of the Central Nervous System (CNS) in acute leukemia confers poor prognosis and lower overall survival. Existing CNS-directed therapies are associated with a significant risk of short- or long-term toxicities. Leukemic cells can metabolically adapt and survive in the microenvironment of the CNS. The supporting role of the CNS microenvironment in leukemia progression and dissemination has not received sufficient attention. Understanding the mechanism by which leukemic cells survive in the nutrient-poor and oxygen-deprived CNS microenvironment will lead to the development of more specific and less toxic therapies. Here, we review the current literature regarding the roles of metabolic reprogramming in leukemic cell adhesion and survival in the CNS.

## Introduction

Acute leukemia is characterized by neoplastic proliferation of immature white blood cells, also called blasts, in the bone marrow (BM), which later rapidly disseminate to the blood and other tissues (Colmone et al., 2008; Vardiman et al., 2009; Glait-Santar et al., 2015). Based on the lineage of affected white blood cells, acute leukemia is classified into acute myeloid leukemia (AML) and acute lymphoblastic leukemia (ALL) (Marks et al., 2009; Lyengar and Shimanovsky, 2021). AML is more common in adults and accounts for about 80% of all AML cases. ALL predominantly occurs in children; it comprises about 80% of childhood and 20% of adult ALLs (Lyengar and Shimanovsky, 2021).

Conventional chemotherapy has successfully decreased the mortality rate of patients with acute leukemia (Marks et al., 2009; Rowe and Tallman, 2010; Rubnitz, 2017). However, patients still suffer from refractory disease or relapse, signifying the need for the development of more effective therapies (Hunger et al., 2012; Winter et al., 2018). One of the devastating features of leukemia is the ability of leukemic cells to colonize at secondary sites for tumorigenesis (Valastyan and Weinberg, 2011). Particularly, infiltration of the central nervous system (CNS) by leukemic cells contributes to an increase in leukemia mortality and treatment failure (Basu et al., 2014; Si et al., 2018).

Growing evidence suggests the crucial role of intrinsic and extrinsic factors in modulating leukemic cell survival (Silva et al., 2011; Giambra et al., 2015; Pitt et al., 2015; Moharram et al., 2017; Ribeiro et al., 2018; van der Zwet et al., 2021). Leukemic cells remain in a quiescent state and highly depend on intrinsic survival factors while circulating in the blood (Guan et al., 2003). When leukemic cells enter the homing tissue, the microenvironmental niche provides multiple signaling ques supporting leukemia survival (Ninomiya et al., 2007). Metabolic reprogramming provides cancer cells with a unique flexibility in adapting to a variety of cell-extrinsic and -intrinsic stimuli. These metabolic adaptations govern tumor transformation, proliferation, invasiveness, and resistance to therapy (Hanahan and Weinberg, 2011; DeBerardinis and Chandel, 2016). Otto Warburg was the first to recognize the aberrant metabolic behavior of tumor cells. He postulated that cancer tissues have higher levels of glucose uptake compared to normal tissues and that cancer cells rely primarily on aerobic glycolysis to produce adenosine triphosphate (ATP) (Warburg, 1925; Warburg, 1956a). Understanding the mechanism underlying metabolic reprogramming of cancer cells could provide a venue for defining novel therapeutic targets (Wolpaw and Dang, 2018).

Acute leukemias commonly display an increase in glucose uptake and aerobic glycolysis (Suganuma et al., 2010; Kishton et al., 2016; Matthijssens et al., 2021). In addition, enhanced mitochondrial respiration (OXPHOS) increases the reactive oxygen species (ROS) levels in leukemic cells (Han et al., 2019). However, leukemic cells can compensate for the harmful effects of elevated ROS levels by enhancing the expression of antioxidants, which ultimately restore redox homeostasis (Sabharwal and Schumacker, 2014; Khan et al., 2016). Importantly, under energy crisis conditions, leukemic cells rely on non-glycolytic resources (Lee et al., 2013) such as fatty acid oxidation, amino acid oxidation (*e.g*., methionine, cysteine), and glutaminolysis, which all provide essential intermediates to maintain the Krebs cycle (Tabe et al., 2020). In line, growing evidence suggests that major oncogenic drivers, such as PI3K/Akt/mTOR pathway, MYC, FLT3, and RAS, contribute to metabolic rewiring in leukemic cells (Herranz et al., 2015; Rashkovan and Ferrando, 2019).

Cell adhesion plays a key role in cancer progression and metastasis. Adhesion molecules regulate cancer cell survival, differentiation, proliferation, inflammation, and migration. Alterations in cell-cell and cell-matrix adhesion allow malignant cells to increase their motility and degrade the cell-extracellular matrix (ECM) to enter the blood circulation, followed by dissemination to distant sites (Martin et al., 2013). During this multi-step process, cancer cells induce metabolic rewiring to meet distinct metabolic demands (Nepstad et al., 2018; Wei et al., 2020). Recent studies demonstrated that cancer cell adhesion may either induce or be induced by cell signaling pathways associated with metabolic reprogramming (Sousa et al., 2019). In line, several adhesion molecules were identified as critical regulators of leukemia development and chemoresistance (Jacamo et al., 2014; Fonseca et al., 2018; Scharff et al., 2020a; Gutjahr et al., 2021).

The impact of specific genetic lesions (*e.g.*, *MLL* rearrangements, BCR-ABL), CNS niche, chemokines, cytokines, and growth factors in driving leukemic cells to the CNS and meninges has been extensively reviewed in (Heidari et al., 2016; Gossai and Gordon, 2017; Piovan et al., 2018; Zhou et al., 2019; Lenk et al., 2020; Whiteley et al., 2021). The roles of metabolic reprogramming and cell adhesion in leukemia development and progression have also been discussed elsewhere (Heath et al., 2019; Rashkovan and Ferrando, 2019; Windisch et al., 2019; Härzschel et al., 2020; Scharff et al., 2020a; Di Martino et al., 2021). However, how metabolic reprograming and cell adhesion regulate leukemic cell infiltration to the CNS remains unclear. In this mini review we focus on recent advances toward our understanding of the roles played by metabolic reprogramming and cell adhesion in acute leukemia (ALL, AML) colonization into the CNS.

## Clinical Overview of Central Nervous System Involvement in Acute Leukemia

The CNS is a common extramedullary site for infiltrating ALL cells (Lazarus et al., 2006). CNS involvement is detected either at initial treatment or at relapse. The incidence of CNS in ALL at diagnosis is approximately 5–10%. For ALL patients who have received prophylactic CNS directed chemotherapy, the recurrence of CNS disease is 7–15% (Alakel et al., 2017; Holland et al., 2003; Pinkel and Woo, 1994; Gökbuget and Hoelzer, 1998). Factors associated with CNS-ALL include high white blood cell (WBC) count, hypercellular marrow, and extramedullary infiltration (BLEYER, 1984; Cassileth et al., 1988; Pinkel and Woo, 1994; Cortes, 2001).

CNS infiltration in AML is relatively rare (Galati et al., 2013; Loeb et al., 2016). Approximately 0.6–5% of AML patients present with CNS disease at diagnosis, and 3–15% with CNS relapse (Kouser and Hashmi, 2007; Galati et al., 2013; Alakel et al., 2017). Risk factors involved in AML-CNS include elevated serum lactate dehydrogenase (LDH) levels, increased WBC count, subtypes of myelomonocytic/monoblastic/monocytic leukemias, inversion of chromosome 16, mutations in FLT3 and NPM1, expression of CD56 and 11q23 rearrangements (Holmes et al., 1985; Thompson et al., 1986; Chang et al., 2004; Cheng et al., 2015; Rozovski et al., 2015).

While cytospin-based evaluation of cerebrospinal fluid (CSF) is used for diagnosis of CNS disease/relapse in leukemia (Del Principe et al., 2014), this diagnostic method does not identify patients who have occult CNS involvement (Martínez-Laperche et al., 2013; Bartram et al., 2018; Del Principe et al., 2021). Furthermore, studies showed that CNS prophylaxis (cranial radiation and intrathecal chemotherapy) is associated with various neurological toxicities (Pochedly, 1977). Thus, it is imperative to understand the mechanism underlying CNS involvement to develop more accurate diagnostic tools and novel therapies that will eradicate leukemic cells from the CNS while causing less adverse neurotoxicity.

## Metabolic Reprogramming of Acute Leukemia in the Central Nervous System

**FIGURE 1:**
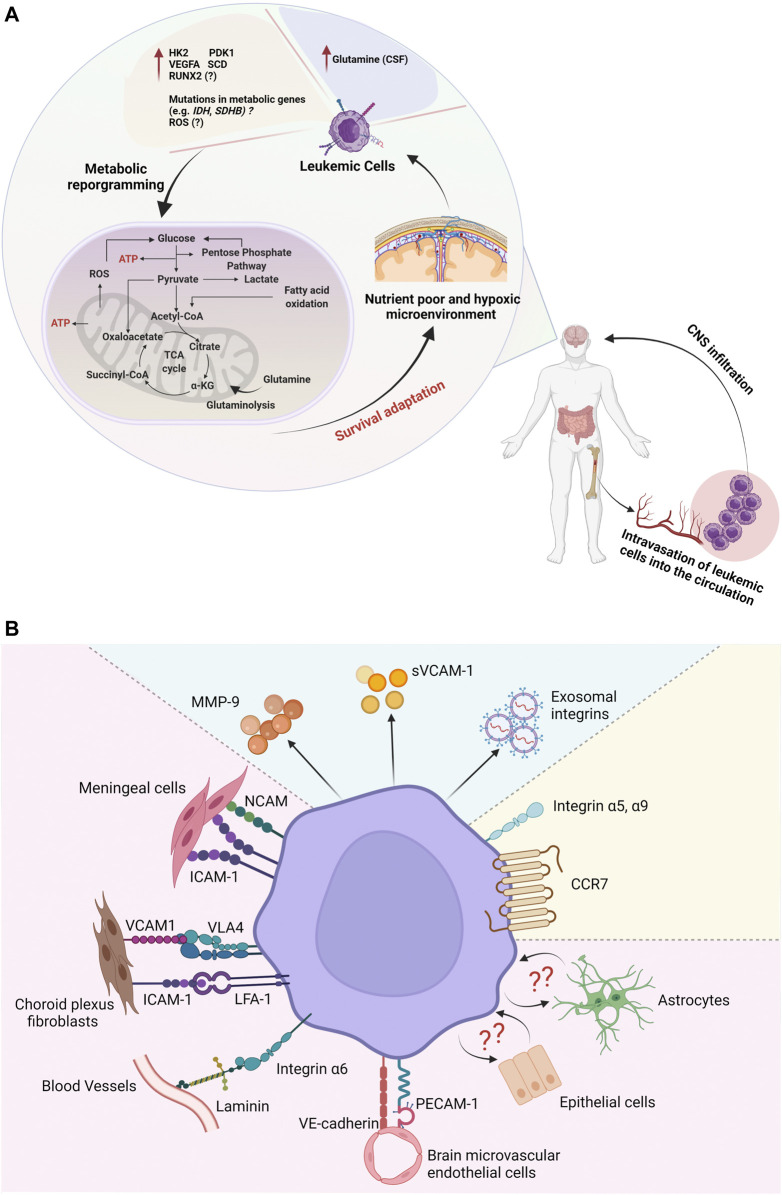
A schematic illustration of metabolic reprogramming and adhesion pathways involved in the central nervous system (CNS) niche in acute leukemia. **(A)** Leukemic cells can adapt to nutrient-poor and oxygen-deprived CNS by modulating the expression of metabolic and hypoxia-associated genes. **(B)** Upregulation of adhesion molecules may facilitate leukemic cell infiltration and survival in the CNS microenvironment.

The CNS serves as a sanctuary site, in which leukemic cells evade the immune response and systemic chemotherapy (Frishman-Levy and Izraeli, 2017). Metabolic adaptation in the CNS niche is a prerequisite for the long-term survival of leukemic cells and the recurrence of the disease (Savino et al., 2020). Leukemic cells depend on cellular metabolism rewiring to survive in nutrient-poor and hypoxic microenvironments (Cairns et al., 2011; Cha and Lee, 2016; DeBerardinis and Chandel, 2016). Thus, metabolic vulnerabilities of leukemic cells could be used for therapeutic purposes (Kuntz et al., 2017; Nachmias and Schimmer, 2018). Despite recent advances in cancer metabolism research, little is known about whether and how cell metabolism affects the migration and adhesion of leukemic cells in the CNS. A better understanding of these metabolic adaptations will advance the development of novel treatment strategies. Below, we discuss major metabolic pathways and their roles in leukemia colonization of the CNS (Figure 1A).

### Glycolysis

Glycolysis takes place within a cell’s cytosol fraction in the presence (aerobic) or absence (anaerobic) of oxygen. Under anaerobic conditions, lactate is the final product of glycolysis, in which two adenosine triphosphates (ATP) are formed. In aerobic conditions, a glucose molecule is transformed into two pyruvate molecules, which are processed into lactate or enter into the Krebs cycle. This process generates 4 ATP and 2 nicotinamide adenine dinucleotide hydrogen (NADH) molecules (Allard et al., 1994).

Strong evidence suggests that leukemic cells have increased glycolysis (Warburg, 1956b; Boag et al., 2006; Herst et al., 2011; Calviño et al., 2014; Liu et al., 2014; Poulain et al., 2017; Robinson et al., 2020; Matthijssens et al., 2021). In line with this, Kato et al. (2017) compared the transcriptome of B-cell acute lymphoblastic leukemia (B-ALL) cells derived from the CNS and BM of xenografted mice, and the BM and CSF of pediatric B-ALL patients with CNS disease. CNS-derived leukemic cells adapted to hypoxic conditions by upregulating the genes associated with hypoxia such as hexokinase-2 (HK2), pyruvate dehydrogenase kinase 1 (PDK1), and vascular endothelial growth factor A (VEGFA) whereas genes associated with the cell cycle and oxidative phosphorylation were downregulated (Kato et al., 2017). Interestingly, VEGF mediated B-ALL cell entry and infiltration into the leptomeninges. However, the potential link between VEGF and glycolysis in leukemic infiltration of the meninges has not yet been established.

We recently reported that Runt-related transcription factor 2 (RUNX2) was upregulated in children, adolescents, and young adults with high-risk T-ALL and its increased expression was associated with leukemic cell migration and dissemination of T-ALL to extramedullary sites including the meninges (Matthijssens et al., 2021). RUNX2 potentiated T-ALL metabolic activity by enhancing ATP production and glycolysis *in vitro*. Specifically, RUNX2 induced LDHA, PGK1, and GLUT1 expression concomitant with an increase in glucose uptake and glycolysis. Treatment with 2DG, an inhibitor of glucose metabolism (hexokinase inhibitor), reduced T-ALL migration, indicating a potential role of glycolysis in RUNX2-mediated T-ALL cell chemotaxis (Matthijssens et al., 2021). Further studies are required to determine whether and how glycolysis affects the ability of leukemic cells to invade and survive in the CNS.

### Pentose Phosphate Pathway

The Pentose Phosphate Pathway (PPP) is an alternative branch of glycolysis. It links glycolysis with the production of ribose and NADPH. The PPP comprises the oxidative and nonoxidative phases. In cancer cells, the oxidative phase is involved in maintaining the redox balance in rapidly proliferating cells (Xu et al., 2009). The non-oxidative phase allows different glycolytic intermediates to enter PPP. Studies showed that cancer cells modify PPP for survival and proliferation (Stincone et al., 2015; Bhanot et al., 2017). PPP generates pentose phosphate and NADPH, which are vital for lipid synthesis and cell survival under stress conditions (Riganti et al., 2012; Zhang et al., 2014; Lucarelli et al., 2015; Stincone et al., 2015). However, the role of PPP in leukemia infiltration to the CNS has not been elucidated thus far.

### Krebs Cycle and Amino Acid Metabolism

The Krebs cycle, also known as the citric acid or tricarboxylic acid (TCA) cycle is a central pathway for sugar, lipid, and amino acid metabolism. The Krebs cycle produces building blocks in macromolecular synthesis together with the energy and electron acceptors that are used in downstream cellular processes such as electron transport chain (ETC) reactions. The aberrant function of the TCA cycle has been seen in a wide array of diseases (Jacque et al., 2015; Anderson et al., 2018). Succinate dehydrogenase (SDH) is the enzymatic complex responsible for oxidizing succinate into fumarate. Interestingly, recurring mutations in the *SDHB* gene were identified in T-ALL cell lines and primary pediatric T-ALL samples. These mutations were associated with increased survival of T-ALL cells under hypoxia (Baysal, 2007). In the TCA cycle, isocitrate dehydrogenase (IDH) (Chaturvedi et al., 2013) catalyzes the reversible conversion of isocitrate to alpha-ketoglutarate (*α*-KG) (Haselbeck and McAlister-Henn, 1993). Mutations in IDH resulting in a neomorphic enzyme that converts α-KG to the oncometabolite R-2-hydroxyglutarate (R-2-HG) were found in ∼20% of adults AML (Figueroa et al., 2010; Ward et al., 2010; Chou et al., 2011; Fathi et al., 2012; Chaturvedi et al., 2013). The aberrant accumulation of R-2-HG was shown to promote leukemia development (Chaturvedi et al., 2013). While studies on the roles of *SDHB* and *IDH* mutations in CNS leukemia are still lacking, it seems plausible to speculate that mutations in metabolic genes may contribute to leukemic adaptation to the CNS niche.

Our group reported upregulation of RUNX2 in primary T-ALL harboring *KMT2A*-rearrangements and immature/ETP phenotype. RUNX2 increased both, glycolytic and oxidative metabolism as well as the expression of critical regulators of mitochondrial dynamics and biogenesis in T-ALL cell lines (Matthijssens et al., 2021). Upregulation of RUNX2 increased metabolic potential of T-ALL cells and accelerated T-ALL progression and dissemination to the meninges as well as other organs. The role of the TCA cycle in mediating CNS colonization by leukemic cells has yet to be determined.

Amino acid metabolism is involved in protein and non-protein biosynthesis. Abnormalities in amino acid metabolism have been reported in a variety of cancers, including leukemia (Matre et al., 2016; Musharraf et al., 2017; Raffel et al., 2017; Jones et al., 2018; More et al., 2018; Gregory et al., 2019). Interestingly, children with ALL and associated CNS disease had higher levels of glutamine in CSF relative to patients without CNS involvement. Thus, high levels of glutamine were proposed as indicative of CNS leukemia (Peng et al., 2005). Further investigation is required to determine the roles of amino acid metabolism in leukemic colonization of the CNS.

### Reactive Oxygen Species

The organelles involved in the production of ROS are mitochondria (through electron transport), peroxisomes (*β*-oxidation of fatty acids), and the endoplasmic reticulum (*via* oxidation of proteins). ROS levels were elevated in both chronic (Ciarcia et al., 2010) and acute leukemias (Battisti et al., 2008; Sallmyr et al., 2008). Elevated ROS levels potentiated glucose uptake and proliferation of AML cells (Hole et al., 2013; Robinson et al., 2021). Leukemic cells extracted from the CSF of ALL-bearing mice showed decreased proliferation and viability due to elevated ROS. Interestingly, co-culture of ALL cells with meningeal cells led to a decrease in ROS production concomitant with increased leukemic cell survival and chemoresistance (Basile et al., 2020). Further studies are warranted to determine whether modulating ROS levels could be exploited therapeutically in targeting CNS involved leukemia.

### Fatty Acid Metabolism

Fatty acid synthesis (FAS) occurs in the cytosol, where acetyl-CoA carboxylase 1 (ACC1) catalyzes acetyl-CoA to malonyl-CoA, which is further used by fatty acid synthase for fatty acid synthesis (FAS). By mitochondrial β-oxidation processes, fatty acids are broken down into acetyl-CoA, which then enters the citric acid cycle to produce ATP. Fatty acids can also be converted into triacylglycerol, phospholipids or cholesterol esters. Growing evidence suggests the importance of fatty acid metabolism in leukemia development and survival (Ito et al., 2021). For instance, Tucci et al. (2021) reported a unique interaction between ALL cells and adipocytes. In the presence of leukemic cells, adipocytes transferred free fatty acids to ALL cells to fuel leukemic cell metabolism and alleviate ALL dependence from *de novo* lipogenesis (Tucci et al., 2021). In line with this, metabolic adaptation was observed in B-ALL cells infiltrating the liver. In response to the hepatic microenvironment, leukemic cells upregulated endothelial lipase, LIPG, which in turn promoted leukemic cell proliferation and survival through the regulation of polyunsaturated fatty-acid metabolism. Furthermore, tissue damage caused by infiltrating leukemic cells induced the release of liver-derived enzymes, which affected stability of chemotherapy drugs and exerted a chemoprotective effect on leukemic cells. (Ye et al., 2021). On the contrary, CSF is poor in fatty acids compared to the plasma. Thus, leukemic cells colonizing the CNS must rely on *de novo* fatty acid synthesis. Interestingly, B-ALL cells derived from CSF of pediatric B-ALL patients with isolated CNS relapse showed increased expression of stearoyl-CoA desaturase (SCD) compared to diagnostic BM samples (van der Velden et al., 2016; Savino et al., 2020). SCD is a central lipogenic enzyme regulating the synthesis of monounsaturated fatty acids. Furthermore, SCD was also upregulated in CNS ALL cells of animals xenografted with primary B-ALL cells and B-ALL cell lines compared to leukemic cells extracted from the BM or spleen. The mice injected with SCD overexpressing cells showed enhanced CNS infiltration relative to control animals pointing to the role of SCD-mediated lipid metabolism in facilitating leukemia adaptation to the CNS niche. (Savino et al., 2020). In addition, patients who presented with isolated CNS relapse had increased expression of SCD in a sub-population of BM-derived B-ALL cells at diagnosis (van der Velden et al., 2016).

## Cell Adhesion in Leukemic Cell Colonization of the Central Nervous System

Leukemic cells regulate the expression of adhesion molecules to confer a pro-survival advantage against chemotherapy and to increase their invasiveness to extramedullary sites (Erbani et al., 2020; Barbier et al., 2020; Wang et al., 2018; Gaynes et al., 2017; Akers et al., 2011). Growing evidence suggests that leukemic cells invade and colonize the leptomeningeal microenvironment through specific adhesion and homing mechanisms (Figure 1B). Co-culture of ALL cell lines and primary B- and T-ALL cells with meningeal cells enhanced leukemic cell survival compared to leukemic cells incubated in the CSF suggesting the importance of leukemic and meningeal cell-cell interactions in promoting ALL cell survival (Basile et al., 2020). In fact, Jonart et al., (2020), demonstrated that ALL cells adhere to meningeal cells and that cell-cell adhesion governs leukemic cell dormancy and resistance to chemotherapy. Importantly, disruption of the meningeal ALL adhesion with tMe6TREN (Tris [2-(dimethylamino)ethyl]amine) increased the efficacy of chemotherapy in the CNS in leukemia xenograft murine models (Jonart et al., 2020). Other studies demonstrated that B-ALL cells from children and B-ALL cell lines were adherent to astrocytes, choroid plexus fibroblasts, and epithelial cells, thus promoting leukemic cell survival and chemoresistance (Akers et al., 2011; Fernández-Sevilla et al., 2020).

Integrins and their ligands play a vital role in leukemic cells migration and homing through mediating cell-cell and cell-ECM adhesion (Scharff et al., 2020a). To date, few studies have identified specific adhesion molecules potentially associated with leukemia infiltration in the CNS/meninges. Increased expression of intercellular adhesion molecule 1 (ICAM-1) was correlated with CNS disease in pediatric ALL samples and B-ALL patient derived xenografts (Mielcarek et al., 1997; Holland et al., 2011). Furthermore, the expression of CD56, a neural cell-adhesion molecule (NCAM), was elevated in adult ALL samples with CNS involvement (Ravandi et al., 2002; Hu et al., 2017). NCAM was also associated with intracerebral and leptomeningeal infiltration in adult T cell leukemia (ATL) (Hashiguchi et al., 2002). Thus, NCAM was proposed as a marker for CNS infiltration and poor prognosis in ALL, and ATL. In addition, AML and ALL patients who had elevated levels of matrix metalloproteinase-9 (MMP-9) and soluble vascular cell adhesion molecule 1 (sVCAM-1) in CSF were at risk of CNS involvement (Si et al., 2015).

Elegant studies by Yao et al.(2018), demonstrated that B-ALL cells invade the CNS along emissary vessels passing between vertebral and calvarial BM, and the subarachnoid space. ALL cells expressed integrin subunit alpha 6 (α6), a laminin receptor, which interacted with laminin expressed on the bridging vessels, thus mediating the migration of ALL cells into the meninges (Yao et al., 2018). ALL xenografts treated with specific α6 integrin-neutralizing antibodies showed reduced leukemia burden in the CSF/meninges. High levels of *Itga6* mRNA (encodes α6) were also found in leukemic cells in a BCR-ABL1-driven murine model of meningeal leukemia (Yu et al., 2019). On the contrary, recent studies identified *ITGA5* (α5) and *ITGA9* (α9) expression positively correlated with CSF colonization in primary B-ALL samples (Scharff et al., 2020a; Scharff et al., 2020b).

Functionally, co-culture of B-ALL Nalm6 cells with choroid plexus fibroblasts resulted in upregulation of VLA-4 and LFA-1 in leukemic cells concomitant with increased expression of relevant integrin ligands, VCAM1 and ICAM1, in the tested fibroblasts. The inhibition of VLA-4/VCAM-1 signaling with anti-VLA-4 antibodies sensitized co-cultured leukemic cells to chemotherapy (Fernández-Sevilla et al., 2020). Further studies are required to determine whether targeting VLA-4/VCAM-1 adhesion could be used to eradicate CNS involved leukemia. Other adherent junction proteins such as VE-cadherin and PECAM-1 increased the adhesion and migration of B-ALL cell lines through the human brain-derived microvascular endothelial cells but their role in CNS leukemia has yet to be determined (Akers et al., 2010). In another study, CCR7 was sufficient to drive leukemic cells to the CNS in T-ALL. Interestingly, gene expression analyses identified deregulation in genes encoding integrins and metalloproteases that could potentially interact with CCR7 function to support T-ALL invasion of the CNS (Buonamici et al., 2009). Moreover, recent studies showed that pediatric ALL-derived exosomes contributed to leukemic cell invasion in a model of the blood-cerebrospinal fluid barrier (BCSFB) *in vitro*. Interestingly, binding/uptake of ALL-derived exosomes was dependent on various exosomal integrins such as *α*V, *α*5, *β*1, and *β*3 (Erb et al., 2020).

## Summary

CNS involvement has been emerging as a major challenge in acute leukemia treatment. Patients with CNS infiltration have a low survival rate, particularly those with recurrent or refractory disease. The interaction between leukemic cells and the CNS microenvironment promotes leukemic cell quiescence and subsequently the resistance to chemotherapy. To date, few studies have investigated the roles of chemokine receptors and other molecules in leukemia trafficking to the CNS (Buonamici et al., 2009; Williams et al., 2014; Jost et al., 2016; Williams et al., 2016; Alsadeq et al., 2017; Oruganti et al., 2017; Alsadeq et al., 2018; Prieto et al., 2018; Lenk et al., 2021). A growing body of evidence demonstrates that leukemic cells employ cell-cell and cell-ECM adhesion to reside and survive in the CNS/meninges. Furthermore, we speculate that metabolic reprogramming could provide leukemic cells with sufficient energy to facilitate the invasion and colonization of the nutrient-poor and hypoxic CNS microenvironment. Accordingly, targeting specific cell adhesion molecules and metabolic pathways could potentially increase treatment efficacy and reduce the toxicity of existing therapies. In fact, there are many drugs targeting metabolism or integrin-targeting drugs under clinical evaluation. However, those drugs have not yet been tested for the treatment of CNS involved leukemia. Moreover, the potential cross-talk between metabolic pathways and cell adhesion remains poorly understood, pointing to several questions: Are modifications in leukemic cell adhesion associated with specific energetic demands? Do cell adhesion mechanisms support metabolic rewiring of leukemic cells in the CNS niche? Do metabolic adaptations of leukemic cells lead to changes in cell-cell and cell-ECM adhesion within the CNS microenvironment? What is the role of meningeal fibroblasts and other cells within the CNS microenvironment in facilitating leukemia infiltration? Do they undergo further metabolic adaptations and/or express specific adhesion molecules to create a more permissive microenvironment for leukemia colonization? Further investigation is needed to uncover the specific adhesion molecules and metabolic adaptations underlying CNS disease and CNS relapse in leukemia.
